# Shaping by Internal Material Frustration: Shifting to Architectural Scale

**DOI:** 10.1002/advs.202102171

**Published:** 2021-10-29

**Authors:** Arielle Blonder, Eran Sharon

**Affiliations:** ^1^ Racah Institute of Physics HUJI The Hebrew University Edmond J. Safra Campus Jerusalem 9190401 Israel

**Keywords:** architecture, fiber composites, frustrated materials, mold‐less fabrication, self‐shaping

## Abstract

Self‐morphing of thin plates could greatly impact the life if used in architectural context. Yet, so far, its realizations are limited to small‐scale structures made of model materials. Here, new fabrication techniques are developed that turn two conventional construction materials—clay and fiber composites (FRP)—into smart, self‐morphing materials, compatible with architectural needs. Controlled experiments verify the quantitative connection between the prescribed small‐scale material structure and the global 3D surface, as predicted by the theory of incompatible elastic sheets. Scaling up of desired structures is demonstrated, including a method that copes with self‐weight effects. Finally, a method for the construction of FRP surfaces with complex curvature distribution is presented, together with a software interface that allows the computation of the 3D surface for a given fiber pattern (the forward problem), as well as the fiber distribution required for a desired 3D shape (the inverse problem). This work shows the feasibility of large‐scale self‐morphing surfaces for architecture.

## Graphical abstract

Self‐shaping by material frustration is demonstrated for the first time at an architectural scale and in structural materials, typical of the construction field. Quantitative comparison with theoretical predictions, as well as scaling‐up and gravitation effect are presented. Together with a flexible design interface, it suggests a future mold‐less architectural application.

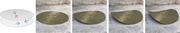

## 1 Introduction

The shaping of matter into complex smooth surfaces as the outcome of internal stresses is abundant in nature. Only recently, it became the subject of various explorations, studying it as model for different morphing mechanisms^[^ ^1^, ^2^, ^3^, ^4^, ^5^, ^6^ ^]^ using different materials that can undergo large inelastic deformations. These studies revealed a huge range of morphological changes, in which initially flat sheets deform into 3D structures, in a quantitatively controlled manner. Most sheets that perform non‐trivial self‐morphing are known to be geometrically frustrated: their internal geometry—the geometry prescribed by the inelastic deformation fields—cannot be realized as a configuration in 3D Euclidean space. As a result, such sheets are residually stressed and their equilibrium configurations are set via nontrivial competition between different energy terms and can vary a lot, depending on the system's parameters. The theory of incompatible elastic sheets successfully predicts the resultant 3D configurations.^[^ ^7^, ^8^, ^9^ ^]^

Beyond its pure scientific importance and its relevance to biological^[^ ^10^, ^11^, ^12^ ^]^ and nanoscale^[^ ^13^, ^14^, ^15^ ^]^ systems, such ability to morph thin plates into complex shapes is appealing to various domains. Specifically, it can have a high impact in architecture and industrial design, where the manufacturing of complex curved surfaces with the investment of minimal energy/effort, is a major challenge.^[^ ^16^ ^]^ So far, however, the self‐morphing of industrial elements has mainly been implemented through small‐scale bilayer structures that undergo uniaxial bending. The full potential of overall shaping into desired complex curvature remains to be explored.

*The* sustainable fabrication of complex‐curved surfaces is a major challenge in architecture.^[^ ^17^ ^]^ Wishing to reduce the impact of mold‐based fabrication, architectural research is experimenting with alternative formation methods that enhance material capacities, such as form‐active molds ^[^ ^18^, ^19^, ^20^, ^21^ ^]^ and active bending.^[^ ^22^, ^23^ ^]^ Furthermore, the idea of an off‐site fabrication of flat elements that take form rapidly once transported and positioned on site, is appealing for its efficiency in transportation volume and on‐site time management. Inscribed in this general effort, we explore the possibility of self‐morphing by geometrical frustration, at the architectural scale.

Switching from the lab table to the architectural site, we need to address three main aspects: the change of material, change of scale, and the necessity of an adapted design tool.

### 1.1 Materials

The theory of incompatible sheets was demonstrated and developed within the lab‐environment, with “model materials” such as gels ^[^ ^1^, ^6^, ^24^, ^25^, ^26^ ^]^ elastomers^[^ ^27^ ^]^ or nematic elastomers.^[^ ^28^ ^]^Criteria that relate to the architectural world, such as durability, robustness, strength and stiffness, standard dimensions, or cost, are naturally not met by the typical lab materials. The transfer to materials that are suitable for the architectural environment is therefore essential and stands at the core of this research.

### 1.2 Scale

The experiments realized so far in the field of incompatible sheets were aimed at verification and demonstration of theory, or the realization of small objects, ranging from the nanometric molecular scale, to several centimeters. Moving on to the architectural world requires a global scale‐up to the size of meters. Along with questions of shape coherence across scales, scaling up introduces the concern for gravitational energy, so far not taken into account in theoretical models that consider only the elastic energy.

### 1.3 Design Tool

The wish to experiment with material systems that would serve in the hands of architects and designers necessitates an interface where theory is translated into a design tool. While physical simulation tools exist, these do not naturally serve the design community, and its wish for the generation of complex shapes. The development of a design interface, where complex surfaces can be created based on theory, is therefore essential in the shift to an architectural realm.

In this work, we present an unusual track of research: rather than expanding the study of self‐morphing, by developing and using “ideal” laboratory materials, we show how the principles of shaping by frustration can be quantitatively implemented with architecture‐oriented structural materials; we chose ceramics and fiber composites, for the change of volume these undergo in their forming processes and their typical use as thin surfaces (e.g., for architectural cladding). We present different types of morphing of the two materials, demonstrating the quantitative connection between the prescribed material's internal structure and the resultant 3D shapes. We show how such methods can be scaled up to architectural dimensions, taking a standard prefabricated cladding panel as a reference. Finally, we demonstrate a design interface using 3D modeling parametric design platform (Rhino Grasshopper), bridging between the scientific and architectural disciplines.

## 2 The Mechanics of Frustrated Sheets

The theory of incompatible elastic sheets, allows computation of equilibrium configurations of thin plates and shells that are residually stressed. The formalism uses the notion of reference geometry of the sheet—the geometry that is locally prescribed by the rest lengths of the material. The reference geometry within the plane of the sheet is encoded by the reference metric field $\bar{a}$, and the reference geometry associated with variations across the thickness of the sheet is encoded by the reference curvature field $\bar{b}$. The actual metric and curvature fields, *a* and *b*, of any existing surface must fulfill three differential equations, known as the Gauss‐Mainardi‐Codazzi equations (GMC). However, expressing only an intrinsic geometry, $\bar{a}$ and $\bar{b}$ can violate these equations. In such cases, we say that, $\bar{a}$ and $\bar{b}$ are incompatible and there cannot be a configuration, in which $a\;=\bar{a}\;$ and $b\;=\bar{b}\;$. Such sheets are geometrically frustrated (we will use this expression throughout the text to describe this type of sheets). Since any deviation of the actual geometry from reference geometry indicates an elastic strain, geometrically frustrated sheets are residually stressed, containing stretching (due to $a\neq \bar{a}$) or bending (due to $b\neq \bar{b}$) energies and their 3D configuration is set by the competition between these two energies. The outcome of this competition, i.e., the 3D shape, depends on the reference geometry, but also on different parameters, such as lateral dimensions and thickness of the sheet.

Local deviations of the actual metric from the reference one indicate in‐plane strain, which leads to stretching energy density of the form: $\varepsilon_{s}\propto t{\left(a-\bar{a}\right)}^{2}$, where *t* is the sheet thickness. Similarly, local deviations of the actual curvature from the reference one lead to bending energy density of the form: $\varepsilon_{B}\propto t^{3}{\left(b-\bar{b}\right)}^{2}$. The total elastic energy of the sheet is a surface integral of these energy densities, where *ds* is a surface element:

(1) $$E\;=E_{S}\;+E_{B}\propto t\int {\left(a-\bar{a}\right)}^{2}ds+t^{3}\int {\left(b-\bar{b}\right)}^{2}ds$$

Minimizing this energy, by selecting the optimal *a* and *b* for given $\bar{a}$ and $\bar{b}$, determines the equilibrium configuration. Two important points should be noted. The different scaling of the stretching and bending energies with the thickness (*t* and *t* ^3^ respectively) implies that in the thin limit, *t* → 0, in‐plane strain must vanish. This implies that in equilibrium configurations we have $a\approx \bar{a}$, i.e., the system “obeys” the reference metric. The other, thick limit, is not well defined, but qualitatively, thick sheets “obey” the reference curvature, $b\approx \bar{b},\;$sustaining in‐plane strain.

One can distinguish between two basic types of geometrically frustrated sheets. Non‐Euclidean Plates (NEP) have zero reference curvature, but their reference metric is non‐Euclidean, i.e., prescribes (via Gauss's Theorema Egregium) nonzero Gaussian curvature. Another type of frustrated sheets is incompatible shells. These have non‐zero reference curvature and an incompatible reference metric. The reference curvature in such systems stems from variations in the inelastic strain, *ε*, across the thickness *t*. The typical magnitude of the reference principal curvatures is $\left|k\right|\approx \frac{\varepsilon }{t}$. Incompatible shells with Euclidean reference metric and isotropic,^[^ ^13^, ^27^ ^]^ or saddle‐like ^[^ ^29^, ^30^, ^31^ ^]^ reference curvature, in either disc or ribbon geometries, were studied. Such systems demonstrate a sharp shape transition, between the curvature‐dominated regime and the metric‐dominated regime. The relevant dimensionless parameter that governs this transition is $\tilde{W}\equiv W\sqrt{\frac{k}{t}}\approx \frac{W}{t}\sqrt{A\varepsilon }$, where *W* is the disc diameter, or ribbon width, *ε* is the inelastic strain and *A* is a geometrically‐determined numerical pre‐factor, of order unity. Ribbons with $\tilde{W}<1$ are governed by the reference curvature and attain double curvature configurations. For $\tilde{W}>1$, the Euclidean metric dominates, and the configurations are developable, i.e., have zero Gaussian curvature. Such configurations typically realize one of the principal curvatures, while eliminating the other.^[^ ^29^ ^]^ The transition between the two regimes is sharp, spanning a range of ≈ 0.5 in $\tilde{W}$. For a sheet with an arbitrary shape, $\tilde{W}$ is determined by the smaller lateral dimension, that will be marked *W* throughout the text. The bending‐stretching transition takes place at a critical value of $\tilde{W}$, of order unity. The specific critical value should be computed for each specific boundary shape.

Principles of the same type are expected to hold in more complex combinations of incompatible reference curvature and metric fields and lead to, yet unexplored, wide range of geometrical and mechanical performances, thus providing guidelines for rational design of self‐morphing surfaces.

So far, the study of frustrated sheets aimed at developing the basic understanding of their shaping. It was, thus, limited to materials with isotropic elastic properties, and ignored external effects, such as gravity. When considering anisotropic elasticity, one needs to plug the proper elastic tensor into Equation (1). Anisotropic elasticity is expected to affect the details of the solutions, but not to change the principles of shape selection.

The effect of gravity, which is extremely important for architectural application was not yet studied as well. So far, equilibrium configurations were selected by balancing only the elastic energy terms. When aiming at scaling‐up a desired configuration by a factor *λ*, one needs to account for both the proper determination of the reference geometry, and to make sure that the elasticity is dominant compared to gravity. The first topic is easily handled via dimensional analysis, which implies that the reference curvature (with units of $\frac{1}{\mathrm{length}}$) should be scaled inversely to the scaling factor, $\bar{b}∼\lambda^{-1}$ and the reference Gaussian curvature should be scaled like $\bar{K}∼\lambda^{-2}$ .

As for the second issue, we note that the gravitation energy density of a structure of height *A*, density *ρ*, elastic modulus *Y* and thickness *t*, scales as *tρgA*, while the elastic energy scales as *Yt* ^3^ *k* ^2^ . For a curved sheet of lateral scale *l*, thickness t, density *ρ* and elastic modulus *Y*, the ratio between gravity and elastic energies is $\frac{U_{\mathrm{gr}}}{U_{\mathrm{El}}}∼\frac{\rho gl^{3}}{Yt^{2}}$. This shows that the role of gravity increases with the scale of the structure. Scaling up from the centimeter to the meter scales could, therefore, be a non‐trivial task. However, to some extent, the two aspects of scaling up are partially complemented: If, upon scaling up by a factor *λ*, the thickness is increased by the same factor, keeping *ε* constant, we achieve the desired, $\bar{b}∼\lambda^{-1}$ relation, while increasing the bending stiffness by a factor *λ* ^3^, which partially solves the problem of self‐weight.

## 3 Materials: Frustrated Composites (FC), Frustrated Ceramics (FCC)

The material families chosen for the research are age‐old malleable ceramics and advanced fiber‐based polymer composite (FRP). The ceramic material family has been in architectural use since ancient times, from hand‐made mud bricks to contemporary cladding solutions or intricate casting.^[^ ^32^ ^]^ Fiber composites are applied today as advanced solutions for elements requiring high performance, complex morphologies or unique surface finishes.^[^ ^33^, ^34^ ^]^ Traditionally, fabrication processes of both material families are mold based.^[^ ^35^ ^]^ Here, we develop alternative techniques that allow for mold‐free shaping of these materials.

Both material families achieve their final constitution through heating (heat curing of FRP and firing of the ceramics), an irreversible transition during which a critical process of shrinkage takes place. In both practices, shrinkage is well known and considered carefully in the design and engineering of the finished artifact, aiming to minimize its undesired effect. In clay, a linear shrinkage of 3–15% of the original material is typical, depending on material type and its firing procedure. In order to avoid the build‐up of internal stresses that would generate cracks or local weakness of the material, best (traditional) practice avoids combining clays with highly different shrinkage ratios.^[^ ^36^ ^]^ In FRP, the shrinkage is uniaxial in a perpendicular direction to fiber orientation, as commonly used polymers typically shrink by 3 to 10% and fiber shrinkage is negligible. This strong effect of fiber orientation generates distortions, traditionally regarded as undesired and avoided by the design of laminates, which are symmetric in their section, to balance and eliminate internal torques.^[^ ^37^ ^]^

**Figure 1:**
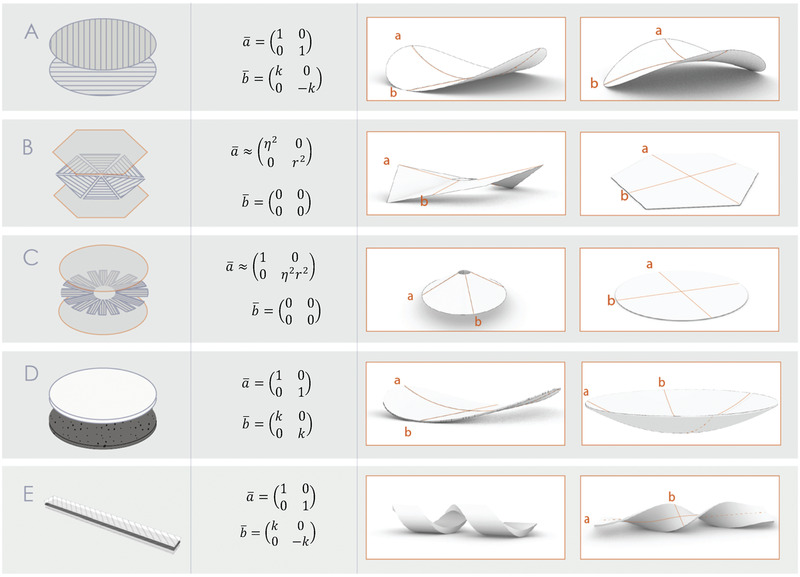
A schematic catalog of possible geometries in frustrated ceramics and frustrated composites, showing the material construction (left column) the reference metrics and reference curvature (middle column), and resulting configurations (right column), within the different regimes: stretching‐dominated (left) and bending‐dominated regime (right). (see physical examples in Figure S3 in the Supporting Information). Frustrated composites A) incompatible shells made by two layers of unidirectional fibers. B,C) Non‐Euclidean Plates, made by one layer of patterned unidirectional fibers sandwiched in two isotropic layers of epoxy. Frustrated ceramics: D) Incompatible shells made by two joint layers of high‐shrinkage ceramic (white) and low shrinkage ceramic (dark). E) Triple‐layered construction, low shrinkage ceramic sandwiched between two layers of higher shrinkage ceramic with opposing grooves, resulting in twisted ribbons.

Here, we harness the shrinking phenomenon and its subsequent internal stresses for the generation of complex morphologies, through frustrated composites (FC) and frustrated ceramics (FCC). Therefore, instead of minimizing or eliminating the effects of differential shrinkage, we wish to enhance it. Based on the theory of incompatible sheets, different material constructions in either ceramics or FRP can be classified as non‐Euclidean plates or incompatible shells and generate controlled material frustration, resulting in a variety of predictable morphologies (**Figure** **1**) (see experimental realizations in Figure S3, Supporting Information). In difference with typical model materials, such as gels or nematic elastomers, the shaping processes of these material systems are not reversible, as these are obtained through curing (thermoset polymer resin for FC, and the firing of clay for FCC).

A single layer of composite contracts in the direction perpendicular to fiber orientation, making it similar to sheets commonly found in plant tissues ^[^ ^38^ ^]^ and recently produced in material such as oriented nematic elastomers ^[^ ^28^, ^39^, ^40^ ^]^ anisotropic gels^[^ ^6^, ^30^ ^]^ or stretched polymers.^[^ ^41^ ^]^ Attaching two layers with perpendicular fiber orientations results in an incompatible shell with saddle‐like reference curvature (Figure 1a). In the bending‐dominated regime ($\tilde{W}<<1$), its configuration is of a saddle; In the stretching dominated regime ($\tilde{W}>>1$), two bi‐stable cylindrical shapes are possible, each manifesting one of the principal reference curvatures. Material constructions which are symmetrical across the thickness but contain in‐plane variation of fiber orientation will result in NEP, having zero reference curvature (due to up‐down symmetry), but a non‐Euclidean reference metric. Simple, axisymmetric examples, such as cones, when shrinkage is radial (Figure 1b), or anti‐cones, where shrinkage is in circumference are demonstrated (Figure 1c), (Figure S3, Supporting Information).

Frustrated ceramics are uniform in the plane due to the nature of clay considered isotropic, but can be made nonhomogeneous in section by joining sheets made of two different clay types. In this work, we used porcelain, with a shrinkage of 12–15% and stoneware, typically shrinking by 5–7%, to form a bilayer sheet (Figure 1d). The difference in shrinkage of the two materials induces an isotropic, homogeneous positive reference Gaussian curvature. For $\tilde{W}<<1$, dome‐like configurations are expected, while for $\tilde{W}>>1$, cylindrical configurations are energetically favorable.^[^ ^27^, ^42^ ^]^ In order to generate reference curvature with negative Gaussian curvature, the isotropy must be broken. We introduce directionality in the ceramic construction by grooving the surface, which allows for strain relaxation perpendicularly to the grooves (in the current study grooving was done manually, but automation of the process, using CNC, is straightforward). Ribbons (or sheets) with saddle‐like (negative Gaussian curvature) reference curvature are obtained by constructing a triple‐layered sandwich construction (porcelain‐stoneware‐porcelain) with grooves in opposite directions in the two porcelain layers (Figure 1e). Such ribbons undergo the “twist‐to‐helical” transition, studied in the context of seed pod opening,^[^ ^30^ ^]^ nematic elastomers,^[^ ^39^ ^]^ and amphiphilic supramolecular assemblies.^[^ ^14^ ^]^

## 4 Results

We start by verifying the validity of the theory of incompatible elastic sheets as a framework for the quantitative description of FC and FCC sheets. Specifically, we test the predictions for variations in shape and shape transitions, as a function of various parameters. Several sets of FC and FCC samples were made and their 3D configurations were measured (see Experimental section) and compared to theoretical predictions. We used simple shapes such as circular discs or squares, in order to allow quantitative comparison.

**Figure 2:**
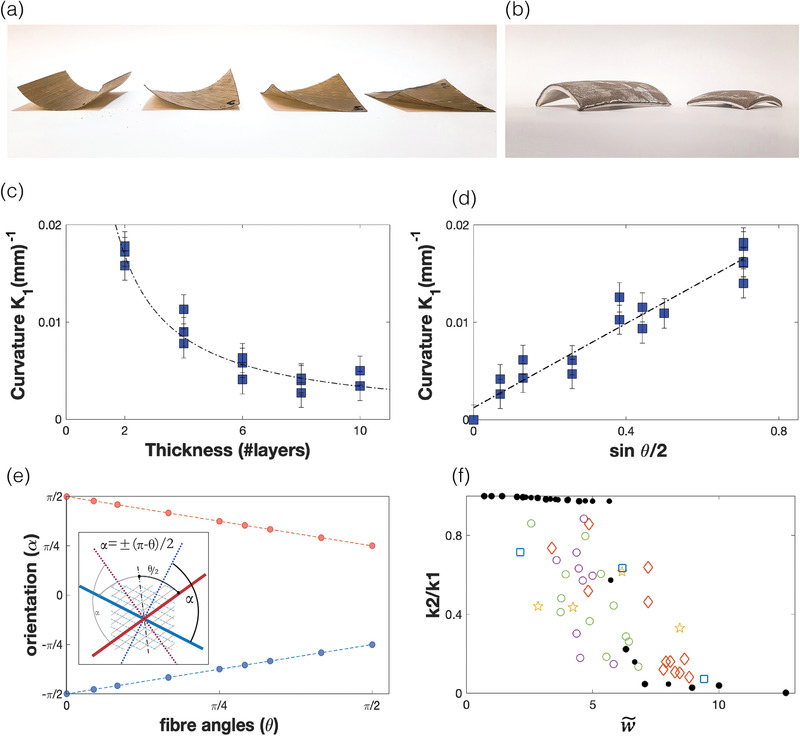
Quantitative relations between curvature and material parameters. a) FC square samples (100 × 100 mm), showing variation in curvature magnitude and orientation according to relative fiber angle between layers, *θ*, (ranging from $\frac{\pi }{2}$ to $\frac{\pi }{6}$) b) FCC square samples (100 × 100 mm,  right;  150 × 150 mm,  left) showing the transition between dome‐like (right) to cylinder‐like (left) shapes, as a function of lateral dimension. c) The measured curvature in a frustrated composite (FC) discs versus the number of layers. The dashed line indicates the expected relation $k\;=\;1.5\frac{\varepsilon }{t}$, with *ε*  =  0.01. d) The measured curvature in FC discs as function of $\frac{\sin\theta }{2}$ (the relative angle between the fibers in the top and bottom layers). The linear dashed line indicates the dependence $k\;=k_{0}\sin\frac{\theta }{2}\;\;$with *k*_0_ as measured in panel c. e) Orientation of measured curvatures, with respect to the bisector of the angle between fiber layers in FC samples (two layers). The measured angles (*α*) follows the relation $\alpha \;=\;\pm \frac{\pi -\theta }{2}$, (dashed lines), i.e., perpendicular to fiber orientations. Inset: an illustration of the relevant angles: the red and blue lines indicate fibers orientations, the dotted lines indicate the directions of principal curvatures. f) The ratio between the small and large principal curvatures, measured in frustrated ceramic (FCC) discs, as a function of $\tilde{W}=\;W\;\sqrt{\frac{k_{0}}{t}}=\frac{w}{t}\;\sqrt{A\varepsilon }$, with *ε*  =  0.2 for the experimental data. FCC‐ critical transition between energy regimes (stretching/bending domination) switching from double curvature to single dominant curvature, at $\tilde{W}\approx 5$. Open symbols showing experimental data and solid black symbols showing simulated data (see Experimental section and Supporting Information).

The first experiment consisted of measuring the magnitude of the curvature for a set of square (100 mm × 100 mm) FC sheets with a varying number (2‐10) of layers. The fibers in the layers (1‐5) at each side of the mid‐plane were in the same orientation, which was perpendicular to the orientation of fibers on the other side. For such lateral dimensions, the FC samples are all within the metric‐dominant (“thin”) regime (Figure 1a). In this regime, only one of the two reference principal curvatures (± *k*_0_) is observed, i.e., *k*_1_≅ ± *k*_0_,   *k*_2_ =  0 and samples are of cylinder‐like shape. Thanks to the flexible nature of the cured material, either stable states of the shape can be realized by reversing of the sheet (“snap through”). The samples were scanned and the principal curvature was measured in the central region of the square (see Experimental section). Plotting the measured principal curvature as a function of the thickness follows the expected trend $k\;=\;k_{0}=\frac{A\varepsilon }{t}\;$ (**Figure** **2**c). Using the pre‐factor *A* = 1.5 (see Equations 4–6 in ref.^[^ ^43^ ^]^), the fit to the data (dashed line) yields a strain value of *ε*  =  0.01 which is in the order of the typical material shrinking rate (3‐5%).

In the second set of experiments, we used circular FC samples (100 mm diameter) made of two layers, with varying angle differences *θ* (0 to 90°) between the fiber orientation of the two layers. Here too, cured samples are all of the cylinder‐like type. Theoretical analysis ^[^ ^29^ ^]^ predicts $k_{0}∼\sin\theta /2$ which is confirmed by our experiments (Figure 2d). The prediction ^[^ ^29^ ^]^ considers materials with isotropic elastic tensor. The elastic properties of FRP are highly anisotropic, with a factor of order 20 between principal stiffnesses. This effect becomes important when considering the orientation of the curvatures as a function of *θ*. For materials with isotropic elastic tensor the actual curvatures are along the reference principal curvatures, which are oriented along $\alpha \;=\;\pm \frac{\pi }{4}\;$with respect to the bisector of the angles between the fibers. In our samples we find $\alpha \;=\;\pm \frac{\pi -\theta }{2}$ (Figure 2e), which implies that the sheet bends perpendicularly to fibers orientations, the direction of lowest bending stiffness and not necessarily along the direction of reference principal curvatures.

As mentioned above, an important characteristic of frustrated doubly curved sheets, is the shape transition, when crossing between bending to stretching dominated regimes. In the first, configurations are doubly curved, while in the second, they are developable, curving along one direction only. For a sheet with lateral dimension *W*, thickness *t* and a relative strain between its top and bottom layers, *ε*, this transition is dominated by the dimensionless number $\tilde{W}\equiv W\sqrt{\frac{\kappa }{t}}\;$=$\frac{W}{t}\;\sqrt{A\varepsilon }$, where *A*  =  1.5, as mentioned above. Isotropic, doubly curved configurations are predicted to appear when $\tilde{W}<<1$, while developable, uniaxially curved configurations are expected for $\tilde{W}\gg 1$. For FC samples, the transition is expected at disc diameter of ∼10mm, excluding the possibility for experimental demonstration of the transition; for this purpose, we used FCC samples.

We prepared several sets of ceramic samples including discs and squares, in various sizes (30 to 100 mm) and thicknesses (4.5 to 11 mm), using two different clay types. The fired samples are dome‐like segments (*K* > 0) or cylinder‐like (*K*  =  0). The samples were scanned and the two principal curvatures were measured in the central region of the discs (see Experimental section). The ratio between the measured principal curvatures, $\frac{\kappa_{2}}{\kappa_{1}}$, can serve as a good indicator of the bending dominated– stretching dominated transition. Numerical simulations of 2D discs with isotropic reference curvature and varying thickness show that the transition takes place at $\tilde{W}\approx 5$ (Figure 2f, black symbols) (see also Figure S4, Supporting Information]. Using the relative strain, *ε*, as a free parameter, we plot the experimentally measured values of $\frac{\kappa_{2}}{\kappa_{1}}$ as a function of $\tilde{W}$ [Figure 2f—colored symbols) We find that the value of *ε*  =  0.2 ± 0.07 gives good agreement between the numerical and experimental data. Indeed, below the transition, $\frac{\kappa_{2}}{\kappa_{1}}\approx 1$, as expected for the dome‐like bending dominated configurations. Above it, $\frac{\kappa_{2}}{\kappa_{1}}\ll 1$, i.e., approaching a developable configurations as expected in the stretching dominated regime.

The scatter of the measured data results from variations in the clay batches, as well as environmental and firing conditions.

### 4.1 Scale

Lab experiments in the field of frustrated materials rarely attempt to address scaling‐up or go beyond the size of few centimeters. As an exception stands the stiff shape‐morphing pneumatic structures programmable by patterned inflating channel, demonstrating that manufacturing process is scalable and architectural size structures are within reach.^[^ ^44^ ^]^ Scaling‐up principles are essential to transferring to the architectural domain, which requires a move from the table‐top size of lab experiments, towards the size of dozens of centimeters to meters. As suggested above, scaling up of a configuration of a frustrated elastic sheet poses two challenges: the first is the proper adaptation of the reference geometry, i.e., the reference metric and curvature fields via the adaptation of a proper material structure. The second is the need of stiffening the sheet in order to stabilize it against the action of gravity.

**Figure 3:**
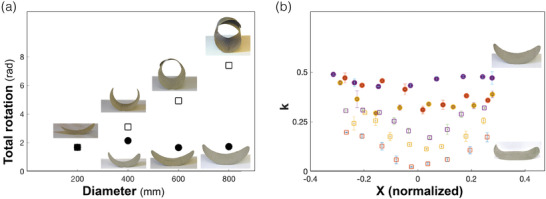
Scaling up and the effect of gravity in FC samples. a) Total surface rotation versus disc diameter measured for free‐hanging discs (negligible gravity effect). Discs with constant thickness of 2 layers (open squares) demonstrate linear increase of total surface rotation, as visible in inset photos. Discs with proportionally varying thickness (solid circles), demonstrate constant surface rotation, i.e., preservation of shape upon scaling. b) The local curvature measured along the principal direction of horizontally placed discs (non‐negligible gravity effect), as function of the normalized distance from the disc's center. While in discs with proportionally varying thickness (solid circles) the curvature is approximately constant, discs of constant thickness of two layers (open squares) flatten in their center, by their own weight. Discs diameters: 800 mm red; 600 mm yellow; 400 mm purple. Inset: images of 800 mm scaled (top) and unscaled (bottom) discs.

In order to address these topics, we prepared two series of saddle‐like FC discs with diameters in the range 200  to800 mm. Such discs expand beyond the typical lab‐scale, of several centimeters, towards the architectural scale (taking an architectural cladding panel as a reference). In one set the discs consisted of two layers, while in the other set, the thickness was scaled up proportionally to the varying diameter. Discs were scanned hanging freely, and principal curvatures were extracted. Integrating the curvature along the disc diameter provides the total rotation of the surface, presented versus disc diameter (**Figure** **3**a). As expected, discs with constant thickness (open squares) had a fixed curvature, leading to an increasing total rotation of the surface. Thus, such sheets did not preserve the shape upon scaling‐up. Contrarily, scaling‐up the thickness leads to the desired decay of the reference curvature as *λ* ^−1^, and therefore diameter‐independent surface rotation (solid circles), i.e., a successful scaling‐up of the shape.

As discussed before, thickening the sheets by a factor *λ* increases their bending stiffness by a factor *λ* ^3^. This outcome helps coping with the second challenge—avoiding distortions and collapse due to self‐weight. In order to measure the effect of gravity, we placed the samples horizontally—an orientation in which the distortion due to gravity is maximal. Measuring the local curvature across discs, shows a clear difference between the two sets of discs. Discs that consist of two layers, undergo a distortion as they are “crushed” under their own weight (Figure 3b, open symbols). The distortion increases as the discs get larger, until most of the central part of the disc is flat. In contrast, for discs with scaled thickness, the curvature is nearly uniform (Figure 3b, solid symbols). These measurements show that scaling up of the entire structure (including thickness), while maintaining material properties (specifically *ε*), properly scales the reference geometry and stabilizes the configuration against the action of gravity. Still, the different scaling of the bending and gravity energies, implies the existence of maximal scale, beyond which, self‐weight significantly affects the configuration. Estimating this scale for the case of an inverted (positioned as in Figure 3, thus unsupported) arc made of FC gives a scale of few dozens of meters (see Section S1 in the Supporting Information).

### 4.2 Design Tool

The design process, by nature, implies the need for a useable design tool that would enable the visualization of the outcome, together with the ability to control it. Currently, there is no platform that allows the design of large‐scale complex frustrated sheets; addressing the architectural realm calls for a design interface for non‐Euclidean frustrated composite sheets (FC). Contemporary architectural applications typically require surfaces with complex distribution of curvature. The realization of such surfaces poses two parallel challenges, of fabrication and of simulation. As demonstrated above, the fabrication methods we presented for FC (with the available commercial materials) rely on the layering of unidirectional sheets that generate surfaces of zero Gaussian curvature. A complex distribution of curvature would require locally oriented placement of fibers. Furthermore, such configurations of complex distribution of curvature cannot be computed analytically, and a numerical platform is thus needed.

Following methods used in liquid crystals elastomers^[^ ^28^ ^]^ and responsive gels,^[^ ^24^ ^]^ we address these challenges by pixelization of the smooth desired surface into square tiles sub‐surfaces of equal size. Each of the tiles is approximated by a surface of constant uniaxial curvature, which can be achieved by a specific fiber orientation within the tile's layers. The global elastic solution of the continuous surface is obtained by the nontrivial energy minimization, and may result in a complex‐curved surface. As the assembly of pixels forms a continuous sheet, the actual curvature of each pixel is affected by its neighboring pixels – both in the amount of curvature and its direction. The isolated pixels can be bi‐stable (with ± *k* as possible stable curvatures) potentially leading to multi‐stability of the complex surface. If certainty with regards to the resulting shape is required, a slight asymmetry can be introduced to favor a required stable state (e.g., additional layers on one side only), or alternatively, initial setting of single curvature, for which $\bar{a}$ and $\bar{b}$ would be compatible, and therefore fully predictable.

**Figure 4:**
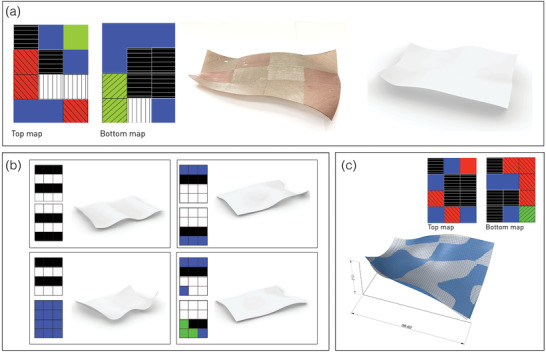
Complex curved panels design and fabrication through pixelization method a) fabrication of a panel according to top and bottom maps with colored tiles indicating fiber orientation (black ‐x aligned; white—y aligned; red and green—± 45°; blue‐ neutral, no fibers). A FC sheet of 800 mm × 600 mm (middle) compared to the simulated surface (right). b) Demonstration of forward‐design process: various generating top and bottom maps (left) and their simulated surface configurations (right) (see experimental realizations in Figure S5, Supporting Information). c) Demonstration of inverse‐design process: target surface was set as the surface obtained in simulation (a). Found surface (blue) compared to target surface (white). The standard deviation between surfaces is 0.53, over a global height of 15.0 (3.5% deviation). Note that the maps of fiber orientations differ from the original one in (a).

We designed a surface of complex curvature, divided into 3  × 4 tiles, each allocated with an axial contraction (0°, 90°,  or ± 45°) by unidirectional fiber orientation, for top and bottom layers (**Figure** **4**a, left). The pixelated panel was fabricated in a scaled size (300 mm  × 400 mm  × 2 layers) and full size (600 mm  × 800 mm  × 4 layers), demonstrating an effective elastic solution that results in a complex‐curved panel (Figure 4a, middle). While still being of medium size, this realization of scaled and large‐sized panels demonstrates the ability to expand beyond the table‐top size towards a full‐sized architectural panel (e.g., 1200 mm  × 2400 mm). As discussed above, this scale is well within the range where gravity is negligible (see Section S1, Supporting Information)

For the numerical simulation of the pixelated surface, we use a commercial readily available 3D software and its parametric design platform (Rhinoceros 3D, grasshopper platform, Kangaroo Physics plug‐in, see Experimental section). The parametric platform incorporates a simulation engine that allows the determination of the properties of a sheet in order to emulate its mechanical properties, and minimizes the elastic energy. In addition, it provides a real‐time visualization, based on simplified physical behavior. Following the fabrication method by surface pixelization, we devised a simple interface where the overall surface is tiled into square sub‐surfaces, comprised of two layers each. Each tile is defined by an axial contraction (along 0°, 90° or ± 45°) for top or bottom layer, allowing the determination of nontrivial $\bar{b}$. In addition, consistently with FC material properties, the axial stiffness along the fibers is set to be 20 times larger than the stiffness in the perpendicular direction. In the examples we present$,\bar{a}$ was selected to be Euclidean and $\bar{b}$ is manipulated. The equilibrium configuration of the complex‐curved surface is obtained via minimization of the elastic energy.

For the validation of the tool, the complex‐curved panel of the physical experiment was simulated; the similarity between configurations of the experimental samples and the simulated one is noticeable (Figure 4a, right), with deviation on the order of 10% (see Figure S6, Supporting Information for comparison between the two surfaces). The ability to generate variations of complex curved surfaces via different patterns of fiber orientation is then demonstrated (Figure 4b) (see Figure S5, Supporting Information for experimental realization of these samples).

This is an interface of a forward design process, where one can design a surface through the variation of fiber patterns of each tile, in a “trial and error” mode, with the instantaneous simulation of the resulting surface. The designer, however, would often require the ability to determine a 3D surface as a target, to be obtained by a suitable fiber pattern, i.e., requiring a process of inverse design. Based on available optimization algorithms of the parametric software (Rhinoceros 3D, grasshopper platform, Galapagos plug‐in, see Experimental section), we devised an interface for the inverse design of a surface, setting it as a target, to be obtained by a fiber pattern that is searched by the engine. For each iteration, the distances between the two surfaces (simulated and target surface) are squared and averaged, to be minimized for an optimal solution. Here we show an approximation to the target surface taken from Figure 4a. We obtain a good approximation to the target surface (Figure 4c), with 0.53 standard deviation between surfaces, over a global height of 15.0 (3.5% deviation). Still, the obtained fiber map differs from the original pattern of target surface (Figure 4a). This demonstrates the high variability in design that results from the large number of controllable degrees of freedom that determine the elastic solution. Since in the inverse design process, the surface configuration, not the fiber orientation, is the target, the existence of multiple solution is a benefit.

## 5 Conclusion

The results presented above show that structural materials, typical of the construction world, can be shaped into complex‐curved elements via the principles of the theory of non‐Euclidean sheets. We developed new protocols of material construction showing that material properties that are undesired in traditional construction techniques, are, in fact, the key for a mold‐less shaping of architectural elements. All the 3D structures presented in this paper, were generated without any mold, or the application of external loads. We presented the primary tools necessary for a major shift in the approach to material shaping for architecture. These include material construction, based on theoretical guidelines, scale‐up principles and finally, computational tools for forward and inverse design of complex surfaces.

In our experiments, we first focused on the existence of tractable quantitative relations that allow for controlled design. We showed that the theory of incompatible elastic sheets is an excellent framework that provides such design principles and relations. As shown (Figure 2), sheets made of, both frustrated composite and ceramics, follow the known behavior of incompatible sheets. The simple relation between the magnitude of curvature and the thickness, or the relative fiber orientation, was demonstrated in FC sheets. The qualitative shape difference between bending dominated and stretching dominated configurations, switching between developable and doubly curved configurations as a function of the disc's diameter, was demonstrated in FCC.

Shifting to new materials introduces new material properties not previously investigated in the domain of NEP, such as relative large thicknesses or extreme anisotropy of material stiffness. These properties do not eliminate the existence of stretching dominated/bending dominated regimes and their qualitative effect on the resulting surfaces. However, they do significantly change quantitative values such as the amount of curvature and its principle directions, make them noticeably different from known results. For example, the bistable principal curvatures in FC discs are found to be perpendicular to fiber's orientation (Figure 2e), in difference with the prediction for isotropic elastic modulus. This is a direct outcome of the large anisotropy of the bending stiffness of FC sheets. This and similar effects can be easily accounted for within the theoretical framework, by using anisotropic elastic tensor. Furthermore, the anisotropy of the material could serve as an additional tool in the hand of the designer, widening the possibilities for shape articulation. Relative and absolute fiber orientation add to the thickness*(t*) and relative strain(*ε*) as handles for surface articulation (Figure 2c–e).

Another challenge we addressed is the scaling of samples up to architecture‐relevant scales. We pointed out that material properties and structure locally determine curvatures (via either $\bar{b}$ or $\bar{a}$). Therefore, when scaling a system up by a factor *λ*, curvatures must be scaled by a factor $\frac{1}{\lambda }$ in order to prescribe the same scaled up 3D shape; this is naturally obtained by thickening the sheet by the same factor *λ*. This was shown useful as a way to account for the effect of self‐weight, related to scaling up. Reducing the reference curvature by thickening the sheet by a factor *λ* increases its bending stiffness by a factor *λ* ^3^. This stiffening partially accounts for the increase in the self‐weight effects that increase as *λ* ^4^. As demonstrated in Figure 3a, indeed a desired fixed configuration can be scaled up and sustain its self‐weight. However, the different scaling of gravitation and elastic energies implies the existence of a typical maximal scale, $L_{\max}\approx \frac{10^{-2}\varepsilon^{2}Y}{\rho g},\;$beyond which, the deformation of the structure under its own weight is larger than1%. It is interesting to note that when gravity *is* significant, configurations are selected via the competition between gravitation and elastic energies, thus potentially extending the range of attainable shapes.

We showed that the architectural need of generating complex smooth surfaces with distributed curvatures, can be addressed through a method of pixelization, which simplifies both design and manufacturing. A parametric design platform commonly used by architects (kangaroo physics plug‐in in Rhino Grasshopper), enables both “forward” design of surfaces, i.e., discovering a shape via manipulation of fiber orientation, and the “inverse” process, in which fiber orientation “map” is found in order to achieve a specific conceived surface. These tools address the designer's need for live feedback in the design process. As shown, the design of such surfaces is characterized by an extremely large number of possible patterns, tunable by various parameters of material construction. These can be used in order to achieve a wide range of configurations, making it a design tool for complex‐curved panels, generated by material frustration.

This work should serve as a proof of principles. It demonstrates the wide space of attainable configurations in both FC (see Figure S1, Supporting Information) and FCC (see Figure S2 in the Supporting Information) material systems. It points to subjects that still need to be studied and optimized. Developing resins for composites with larger shrinkage, a previously undesired property, will increase the range of accessible structures and scales; developing dedicated design software and pushing towards large scale prototypes, are important steps towards turning self‐shaping by frustration into an applicable novel architectural approach.

## 6 Experimental Section

### Material Construction

The FC specimens were prepared with glass/epoxy unidirectional prepreg material, ply thickness 0.3 mm. Specimens were made with the prepreg fabric in an unfrozen state and no additional gluing agents were used between layers. Specimens were made in rectangular shapes of 100 × 100 mm or disc shape of 100 mm diameter, with varying number of layers (2 to 10) according to experiment as detailed (Figure 2). Larger discs were prepared in several diameters between 200 and 800 mm, with varying number of layers (2 to 8) according to experiment as detailed (Figure 3).

The construction of complex curved panels (Figure 4) required an intermediate layer, using 0/90 glass‐epoxy prepreg 300 g m^‐1^. Specimens were made with the prepreg fabric in unfrozen state and no additional gluing agent was used between layers.

Specimens were cured flat in oven of 120 °C, for 2 h. No vacuum or pressure was used. Taken out flat from the oven, the specimens self‐shape upon cooling in air temperature, a process which takes a few dozens of seconds (see Video S1, Supporting Information).

The FCC specimens were prepared with Audrey Blackman porcelain 1220 to 1280° Celsius body 1101, and with stoneware Terrazzo umbra 4020 with 0–2 mm chamotte 40%, by Sibelco.

The FCC specimens are dried and fired in a single continuous firing program of 10 stages, gradually ramping to 1280 °C (see Supporting Information for detailed firing program).

### 6.1 Scanning

3D scanning of FC and FCC specimens (discs and rectangles) was done by profilometer of 1000 × 1000 × 1000 encoder resolution, with a 75 mm lens, at 1 mm density, resulting in points coordinates (*x*, *y*, *z*). Larger FC specimens (Figures 3 and 4) were scanned using Autodesk recap photo application, resulting in STL mesh file, which was then converted to point coordinates (*x*, *y*, *z*) through Rhino3D‐ Grasshopper. For the larger discs, two scans were operated: one free‐hanging scans, where samples were hanging vertically, and another set of scans where samples were placed horizontally on a surface (thus maximizing gravity effect on the disc's surface).

### Numerical Analysis

Point data analyzed in Matlab and surface was approximated by global polynomial fit of second order, from which the two principle curvatures were extracted. A central area of the specimens was measured, proportionally to specimens’ size, to reduce the boundary layer effect.

A set of discs with radius *r*  =  1 and varying thickness *t* and curvature *k* were simulated using a code developed in‐house: the domain was triangulated and the energy of Equation (1) was minimized via gradient descent. Parameters’ values were chosen according to our experiment: thickness (*t)* with values of 0.01 to 0.06 and curvature *k*  =  0.005. The resulting configurations were analyzed by the same process of the experimental data (Figure 2f)

### Design Tool

The design interface was developed in the Grasshopper parametric platform in Rhino3D (McNeel), using the Kangaroo Physics 2.0 plug‐in. With simplified modeling and based on physical principles, the Kangaroo engine approximates an elastic solution of the surface under given conditions, with live visualization and through an accessible interface. Here, the “rod” Kangaroo function was used, translating the fiber pattern of the FC panels into rods of varying contraction values. Axial stiffness was configured accordingly to vary within a range of 1 to 20, following the contraction pattern. The two layers of material were modeled as two‐layered space‐frame comprised of rods of variable contraction (Figure 4).

The inverse‐design code relies on the Galapagos plug‐in for the optimization process, using its simulated‐annealing algorithm. Its fitness criteria were set for minimization of average of squared distances between the target and simulated surfaces.

## Conflict of Interest

The authors declare no conflict of interest.

## Supporting information

Supporting Information

Supporting Information

## Data Availability

Research data are not shared.
