# Supplementary material for: Shaping by Internal Material Frustration: Shifting to Architectural Scale
Source: Adv Sci (Weinh). 2021 Oct 29;8(24):2102171. doi: 10.1002/advs.202102171 (PMC8693067; doi:10.1002/advs.202102171)
Supplement: Supplementary file 1 — Supporting Information [file ADVS-8-2102171-s001.pdf]

WILEY-VCH

© 2021 Wiley-VCH GmbH

ADVANCED SCIENCE
Open Access

Supporting Information

for *Adv. Sci.*, DOI: 10.1002/advs.202102171

Shaping by internal material frustration : shifting to architectural scale

*Arielle Blonder and Eran Sharon**

Supporting Information

Shaping by internal material frustration : shifting to architectural scale

*Arielle Blonder and Eran Sharon**

1. Estimation of maximal stable scale

Here we estimate the maximal lateral scale of an arch made of FC, positioned "horizontally", i.e. with maximal effect of gravity, for which the deformation due to gravity is negligible. We compute the actual curvature of the arc and require a deviation smaller than 1-5% from the reference one:

For a full arc (of π rotation) of length L and thickness t, made of FC we have the following parameters and relations:

A rotation by π implies: $\kappa L = \frac{1.5\varepsilon}{t} L = \pi \Rightarrow t = \frac{L\varepsilon}{2}$

The gravity energy is: $U_{gr} = t\rho g \int_{-\frac{L}{2}}^{\frac{L}{2}} h(l)\, dl \approx 2t\rho g\kappa \int_0^{\frac{L}{2}} l^2\, dl \approx \frac{t\rho g\kappa L^3}{12}$

The elastic (bending) energy is: $\frac{1}{24} t^3 Y(\kappa - \kappa_0)^2 L \approx \frac{1}{12} t^2 Y \varepsilon^2 L$

The derivative of the total energy with respect to κ should vanish:

$$\frac{\partial E}{\partial \kappa} = t^3 Y L(\kappa - \kappa_0) + t\rho g L^3 = 0$$

$$\Rightarrow \Delta\kappa \equiv \kappa_0 - \kappa_{eq} = \frac{\rho g L^2}{t^2 Y}$$

Using $\kappa_0 = \frac{\pi}{L}$ and $t = \frac{L\varepsilon}{2}$ we have: $\frac{\Delta\kappa}{\kappa_0} \approx \frac{\rho g L}{\varepsilon^2 Y}$

Requiring $\frac{\Delta\kappa}{\kappa_0} \leq 0.01$ we get: $L_{max} = \frac{10^{-2}\varepsilon^2 Y}{\rho g}$

Using the FC material properties: $\rho = 1700\ kg/m^3$, $Y \approx 70\ GPa$ and $\varepsilon \approx 0.03$
We find $\boldsymbol{L_{max} \approx 30m}$

2. **Table S1** FCC firing chart

**Step num**	**Temp (Celsius)**	**Duration (Hrs)**
1	40	0.5
2	50	1.0
3	70	0.5
4	70	20.0
5	100	0.5
6	100	2.0
7	500	3.0
8	650	1.5
9	1220	3.0
10	1220	0.25

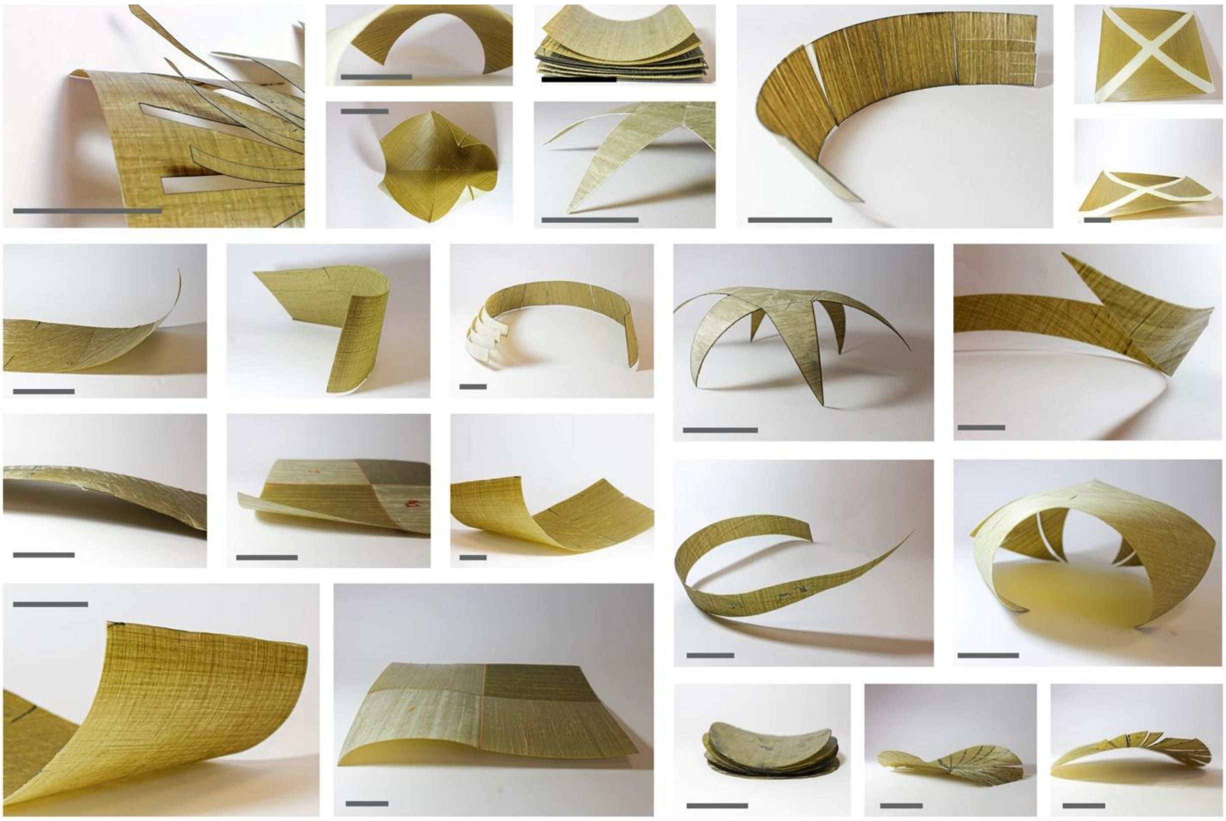

Figure S1 A free demonstration of possible shapes that can be obtained in FC, based on theoretical principles. The shapes, with lateral dimensions ranging between 100-600mm., exhibit a variety of curvature amount, as well as different curvature types. The range of possibilities include frustrated surfaces (double sided patterns) as well as non-frustrated surfaces. Techniques for the generation of curvature variation across the surface are explored, by gradual variation of fiber orientation or by pixelization. The effect of incisions, cuts and patterned boundaries is demonstrated. (All scale bars are 5cm in length).

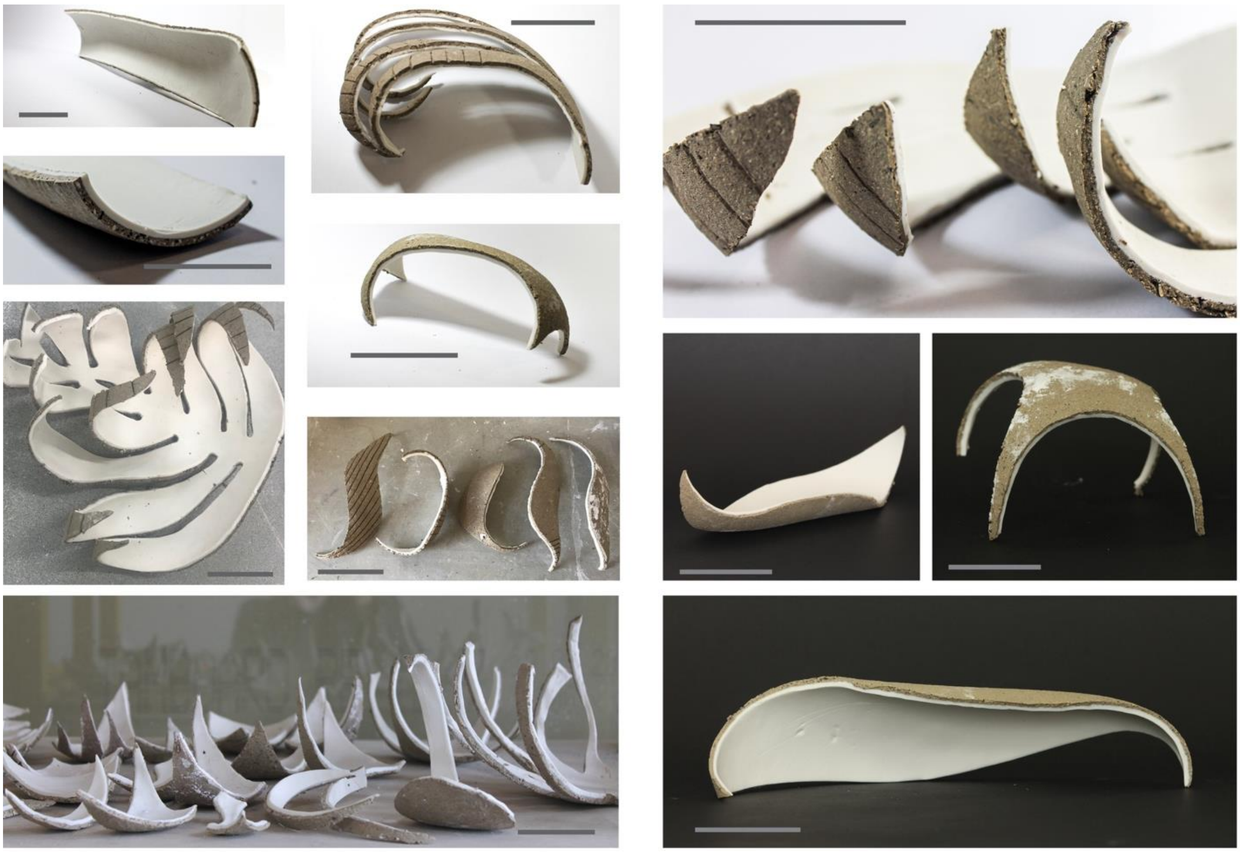

Figure S2 A free demonstration of possible shapes that can be obtained in FCC, based on theoretical principles. The shapes exhibit a variety of curvature amount, as well as different curvature types. In search of significant deformation (z value) and enhanced curvature types. In search of significant deformation (z value) and enhanced curvature, patterned outlines, slender proportions of the 2D shape and the scoring of the bottom terrazzo layer are explored. (All scale bars are 5cm in length).

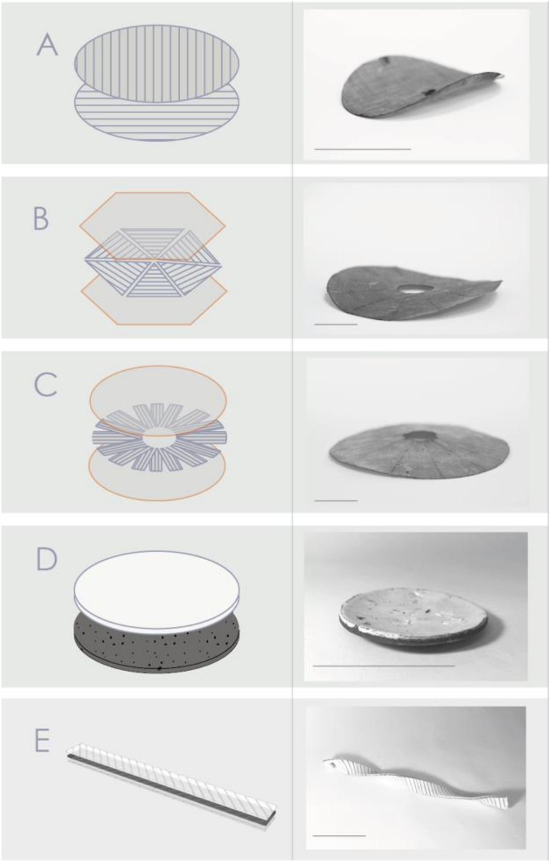

Figure S3 Catalogue of possible geometries in frustrated ceramics and frustrated composites showing material construction (left) and an example of physical realization (right). **Frustrated composites** (A) incompatible shells made by two layers of unidirectional fibers. (B) and (C) Non-Euclidean Plates, made by one layer of patterned unidirectional fibers sandwiched in two isotropic layers of epoxy. **Frustrated ceramics**: (D) Incompatible shells made by two joint layers of high-shrinkage ceramic (white) and low shrinkage ceramic (dark). (E) Triple-layered construction, low shrinkage ceramic sandwiched between two layers of higher shrinkage ceramic with opposing grooves, resulting in twisted ribbons (All scale bars are 5cm in length).

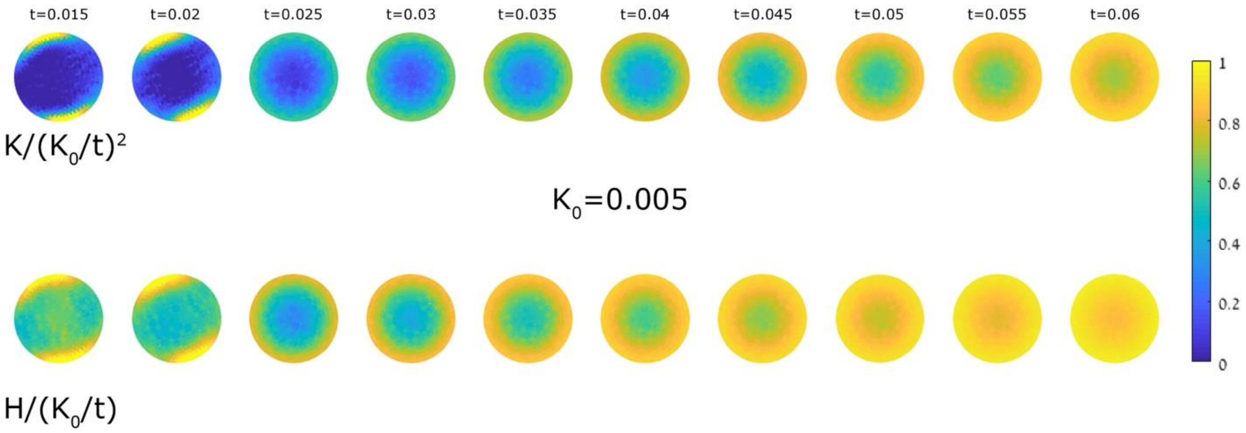

Figure S4 Shape transition in thin/thick regimes: Gaussian (top) and mean (bottom) curvature distribution obtained from numerical simulation of discs of diameter 1 (arbitrary units) and varying thicknesses (t). The transition from single curvature in the thin limit (left) to double curvature in the thick limit (right) is visible

$(K - Actual\ curvature; K\_0 - Gaussian\ curvature; t - Thickness; -Mean\ Curvature)$

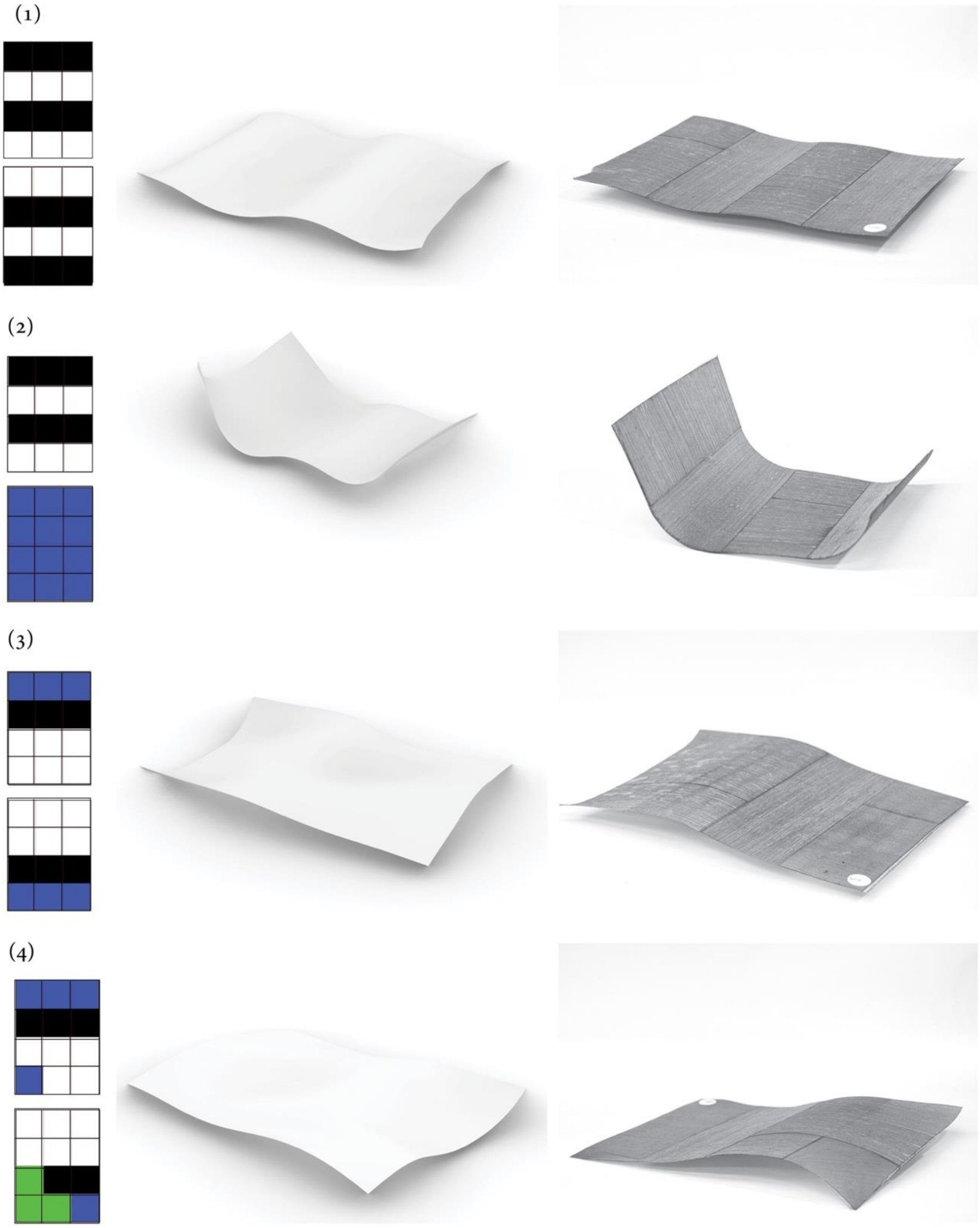

Figure S5 Complex curved panels through pixelization method: Top and bottom maps by colored tiles indicating fiber orientation (black -x aligned; white – y aligned; red and green - $\pm 45°$; blue- neutral, no fibers) (left column); digital simulation (middle column); physical realization, sheet dimensions $300 \times 400\ mm$ (right column);

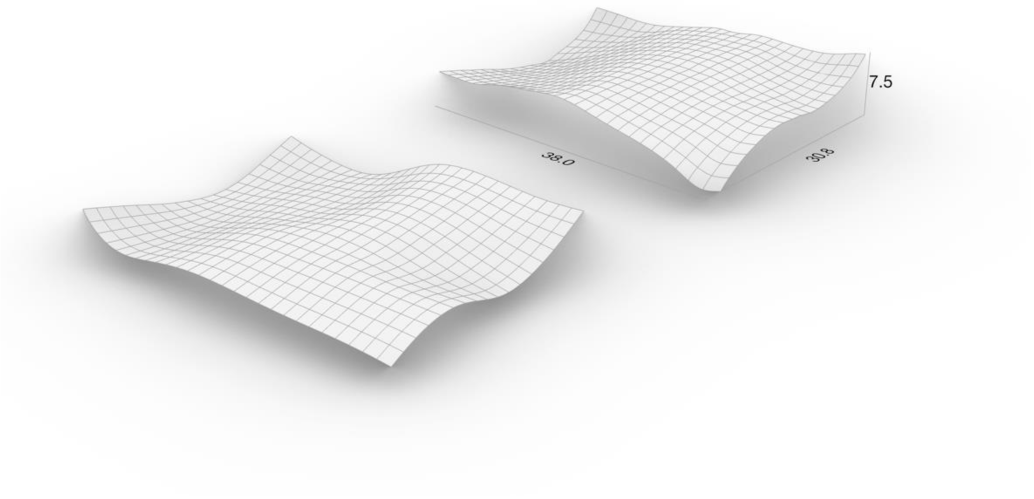

Figure S6 Comparison between simulated (left) and scanned $300 \times 400\ mm$ physical model (right) of complex curved surface. Standard deviation between the surfaces: 0.873cm, over 7.5cm (11.2%).

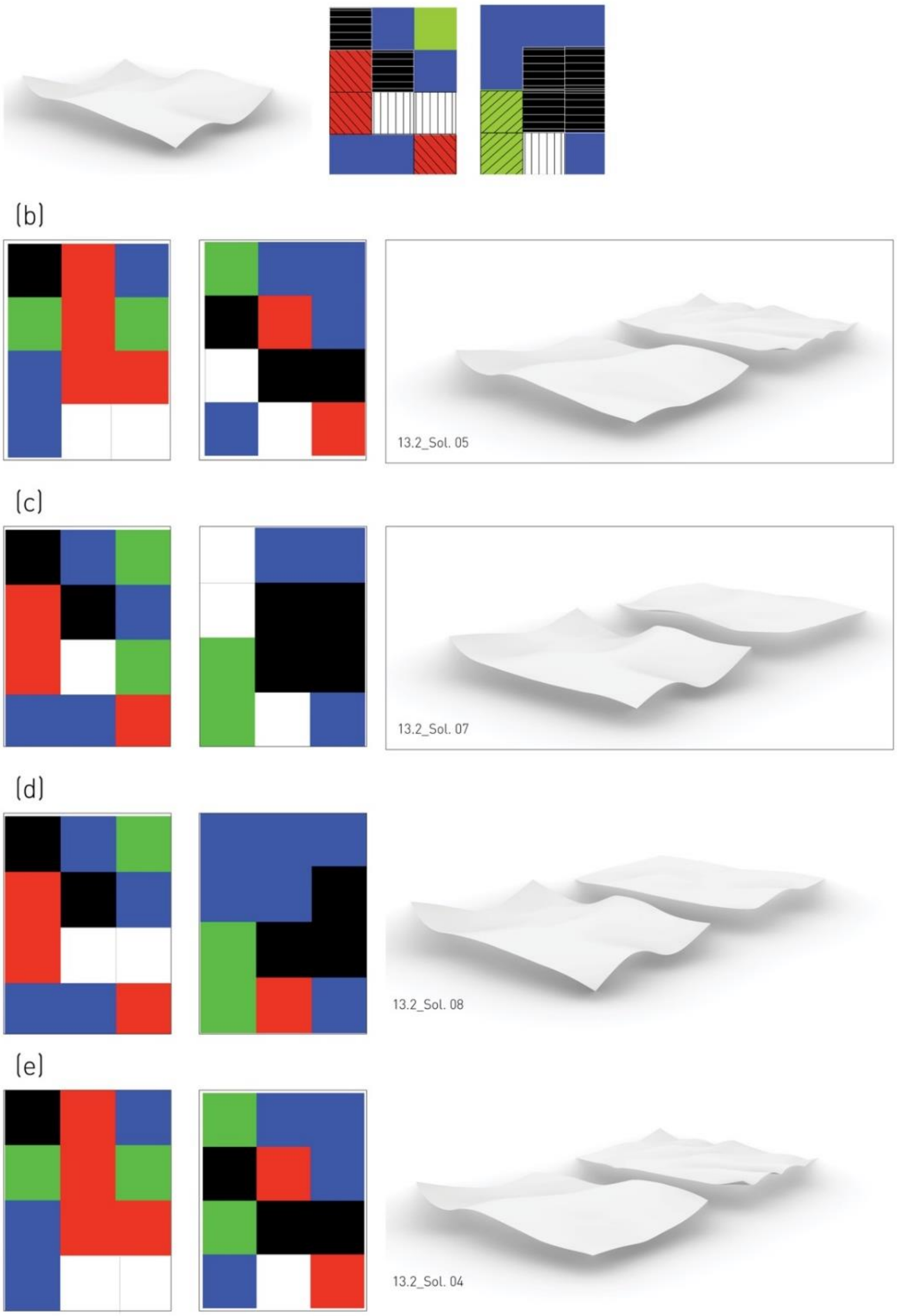

Figure S7 Variants of patterns for a complex panel by pixelization, and their deviation from original surface. Top row : reference panel (identical to the complex panel presented in Figure 4a), its top map (left) and bottom map (right). Patterns (b),(c),(d),(e) are results of the "inverse design" method: top map (left) and bottom map (right), resulting surface (left) and virtual surface of the local differences between resulting and reference surfaces, showing the error amplitude. Average error (as average of local distances (Δz)/ reference surface height amplitude (Z)) : b- 5.96%; c-8.3%; d-5.9%; 6.5% .

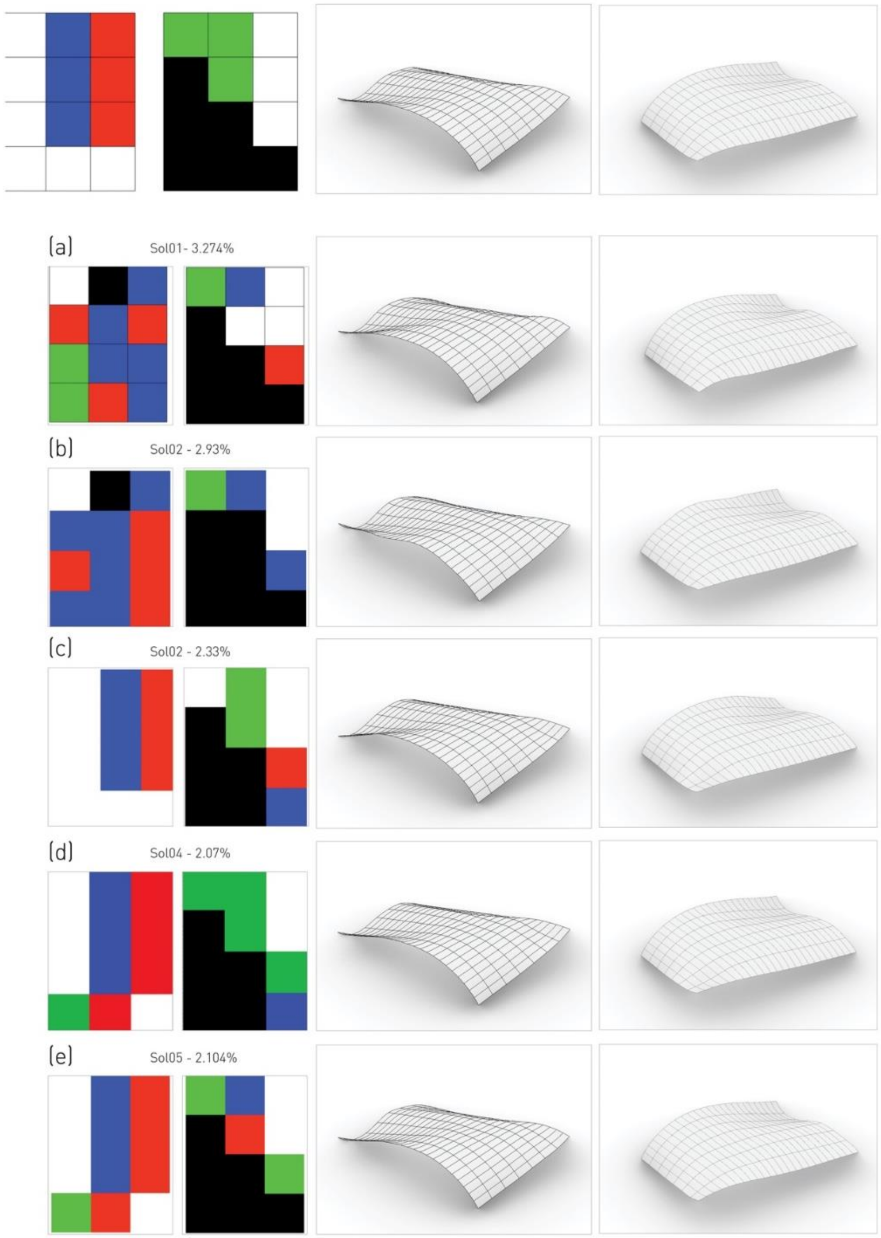

Figure S8 Reverse engineering of complex panel. Top row: reference panel. left: top map, right: bottom map. (a),(b),(c),(d),(e) : solution of searching algorithm (by Galapagos Grasshopper, in Rhino3D platform). For every solution we present: left- top map; right- bottom map and the resultant surface from two different orientations. Average error of found surface to reference surface (as average of local distances (Δz)/ reference surface height amplitude (Z)): a- 3.274%; b-2.93%; c- 2.33%; d-2.07%; e-2.104% (The virtual surface of the local differences is not shown since it appears completely flat)
